# Frequency of missed doses and its effects on the regulation of glucose levels in patients with type 2 diabetes: A retrospective analysis

**DOI:** 10.1097/MD.0000000000037711

**Published:** 2024-04-12

**Authors:** Megumi Shiomi, Tesshu Takada, Katsuya Otori, Kiyoshi Shibuya

**Affiliations:** a Department of Clinical Pharmacy, School of Pharmacy, Kitasato University, Tokyo, Japan; b Department of Pharmacy, Kitasato University Medical Center, Kitamoto, Japan; c Department of Endocrinology, Diabetes, and Metabolism, School of Medicine, Kitasato University, Sagamihara, Japan.

**Keywords:** HbA1c, medication adherence, medication adherence value, pill count, self-adherence of patients, type 2 diabetes mellitus

## Abstract

This study aimed to investigate the association between medication adherence to oral hypoglycemic agents (OHAs) and HbA1c levels in patients with type 2 diabetes mellitus (T2DM) for more than 48 weeks, as well as the factors affecting long-term adherence to OHAs. This retrospective study included 83 patients who had been receiving OHAs for T2DM for ≥48 weeks. Medication adherence values (MAVs) were calculated using the following formula: (total prescription days − prescription days of OHAs brought at admission)/(days from the initiation of OHAs to hospitalization). We assessed the association between HbA1c and MAVs using the Jonckheere–Terpstra test. Furthermore, we examined the association between patient- and medication-related factors and MAVs affecting HbA1c levels. Based on the results, MAVs were categorized as MAV ≤0.86 and MAV >0.86, and factors affecting MAVs were analyzed. Logistic regression analysis revealed that the total number of medications, the number of nonhypoglycemic agents, and a family history of diabetes were independent determinants of MAV ≤0.86 (*P* < .05). Multiple regression analyses indicated that the number of dosages per day and the timing of OHA administration at lunch were independent determinants of lower MAVs (*P* < .05). Our findings suggest that poor medication adherence is associated with elevated HbA1c levels in T2DM patients. Independent factors contributing to poor adherence include a lower number of prescribed medications, fewer nonhypoglycemic agents, no family history, a higher daily dosage frequency, and the administration of OHAs at lunch.

## 1. Introduction

Long-term self-care plays a crucial role in the management of type 2 diabetes mellitus (T2DM). Central to this is adherence to hypoglycemic medications. However, it has been reported that between 36% and 93% of individuals with diabetes take their medications as prescribed.^[1]^ Furthermore, higher HbA1c values are associated with poor adherence to oral hypoglycemic medications (OHAs), higher potential for hospitalization, and increased mortality.^[2,3]^ Therefore, improving medication adherence is a major challenge in diabetes management. Medication adherence can be subjectively or objectively assessed. The average proportion of days covered and medication possession ratio are the main objective parameters used to evaluate OHAs. These measurements are based on information gathered from data on medication claims that record medication usage information billed to insurance companies by pharmacies, and the majority of reports use these measurements as objective measures.^[4–8]^ However, they measured refill behavior only and could not confirm whether the patients actually took their medication, which has been acknowledged as a limitation in previous studies.^[5–8]^ Previous studies using these measures have shown inconsistent results, reporting that various factors are associated with poor adherence, including age, the type of hypoglycemic agent, the number of these agents, and the number of daily dosages.^[9]^ Conversely, pill counting, which is a technique for objective evaluation, has higher accuracy compared to other methods.^[10]^ Pill counting provides more accurate information because the assessment is based on the patient’s remaining medication. However, previous studies on pill counting adherence have described psychological bias and possible concealment by patients as limitations of their studies, since the purpose of the studies was to pill counting.^[11–13]^ Additionally, medication adherence varies depending on who manages and helps patients take medications at home, that is, the patients themselves, their parents, spouses, children, or home care workers. Hence, it is essential to assess medication adherence under the conditions that mitigate this bias. We should investigate patients’ self-adherence with pill counting in situations where patients have less psychological bias. We have previously reported the factors affecting a patient’s medication adherence. In a scenario where the patient’s psychological bias is low, pill counting using medical reconciliation can be performed using an assessment method that we defined as medication adherence values (MAVs).^[14]^ In our previous study, at the time of hospitalization, we included patients who had been taking the same OHAs for more than 24 weeks. However, according to the findings of that study, there was no significant association between HbA1c and medication adherence.^[14]^ For patients with diabetes, maintaining glycemic control with hypoglycemic agents can only be achieved through optimal medication adherence. Therefore, it is important to establish a connection between HbA1c levels and the factors affecting poor medication adherence. Herein, the duration of OHAs was extended to >48 weeks to investigate the impact of medication adherence on HbA1c levels. In addition, the factors affecting low medication adherence were identified.

## 2. Methods

### 2.1. Study design and data source

This study was approved by the ethics committee of Kitasato University Medical Center (registration number H30–009) and was conducted in accordance with the principles of the Declaration of Helsinki. Informed consent was obtained from all participants for the publication of this study. This retrospective study included 83 patients with T2DM who received OHA treatment and were subsequently hospitalized for various treatments at Kitasato University Medical Center between April 2015 and March 2022. Adherence was assessed by counting the OHAs that the patients brought with them at admission. Because the type and frequency of medication consumption could affect adherence, to properly evaluate long-term adherence, only patients who had been taking the same OHAs for at least 48 weeks at the time of admission were included. Patients who stated at the time of admission that they self-managed their medication at home, rather than having it administered by others, such as family members or home health aides, were included.

### 2.2. Participants

#### 2.2.1. Inclusion criteria.

• Patients with T2DM who had received OHA treatment and were subsequently hospitalized for any treatment at Kitasato University Medical Center.
• Patients who could be continuously observed from the initiation of OHA treatment until their hospitalization at Kitasato University Medical Center.
• Patients who had been taking the same OHAs for ≥48 weeks at the time of hospitalization.
• Patients who confirmed that they managed their medication by themselves at home.

#### 2.2.2. Exclusion criteria.

• Patients aged <20 years with visual or other physical impairments, cognitive impairments, mental health diseases, or those who received psychotropic agents.
• Patients with MAV >1.
• Patients who reported the loss of medication or absence of any prescribed medication were also excluded.
• Patients whose OHAs were managed by another person, such as relatives or caregivers at home.

### 2.3. Assessments

We obtained patient characteristics and prescription information for OHAs from medical records. Nephropathy was defined as a urine albumin-to-creatinine ratio of ≥30 mg/g creatinine and/or an eGFR of <30 mL/min/1.73 m^2^.^[15]^ Retinopathy was determined according to the International Clinical Diabetic Retinopathy Severity Scale by a qualified ophthalmologist.^[16]^ We defined retinopathy as mild, nonproliferative diabetic retinopathy or more severe. Macrovascular complications include ischemic heart, cerebrovascular, and peripheral arterial diseases. Chronic diseases are co-existing medical conditions diagnosed by a physician, and patients regularly visit the physician to control the clinical manifestations with or without taking medications. Alcohol consumption was defined as the consumption of alcoholic beverages ≥4 d/wk.

### 2.4. Medication adherence

We calculated the total number of prescription days obtained from the prescription history and the number of OHA prescription days at the time of admission through pill counts.^[14]^ MAVs were determined using the following formula: (total prescription days − prescription days of OHAs brought at admission)/(days from the initiation of taking OHAs to hospitalization).^[14]^ The date of the first filled OHA prescription served as the index date, and the total number of prescription days was calculated from the initial prescription to the last prescription (the last prescription before admission).^[14]^ Patients with an MAV of 1 were those who had not missed any OHA doses from the start of their prescription to the time of hospitalization. As the number of missed doses increased, the MAV decreased, indicating lower adherence. Patients with an MAV >1 were excluded from our study, as they suggested the possibility of medication overdose or incomplete medication brought to the hospital. Additionally, patients who claimed to have forgotten their medications or did not bring them all were also excluded.

### 2.5. Statistical analysis

Data are expressed as median and interquartile range (IQR), frequencies, or percentages. To investigate the impact of MAV on HbA1c, patients were categorized based on their MAVs: ≤0.86 (indicating those who missed taking OHAs on at least 1 day per week), >0.86 ≤ 0.93 (indicating those who missed a day or more every 2 weeks but <1 day per week), and >0.93 (indicating those who missed <1 day every 2 weeks).

We examined the association between MAVs and HbA1c using the Jonckheere–Terpstra test and conducted pairwise comparisons with Bonferroni correction. Based on the relationship between MAVs and HbA1c levels, we categorized MAVs into 2 groups: good and poor adherence. Multiple regression analyses were conducted to explore the factors associated with medication adherence to OHAs. We categorized the factors into patient- and medication-related groups, considering that some patients used multiple types of OHAs. For patients with multiple OHAs, we used the lowest MAV for the patient-related factor analysis. MAVs were treated as dependent variables, whereas patient- or medication-related factors were considered as independent variables. Additionally, based on the medication-related data, we conducted multiple regression analyses with each MAV value as the dependent variable. Variables with *P* < .20 in univariate analysis were included as independent variables. Stepwise regression analysis was used to identify the factors affecting medication adherence. The multicollinearity of variables was assessed using the variance inflation factor (VIF), with serious collinearity defined as VIF ≥5. All statistical analyses were performed using EZR version 1.61 (Saitama Medical Center, Jichi Medical University, Saitama, Japan), a customized version of R Commander designed to incorporate frequently used statistical functions in biostatistics.^[17]^ *P* < .05 were considered statistically significant.

In addition to the abovementioned traditional statistical analyses, the impact of potential predictors on medication adherence was further explored using the Bayesian shrinkage prior models implemented in RStan, which is the R interface Stan. This approach was inspired by Bhattacharyya et al’s work,^[18]^ which discusses the application of shrinkage priors in clinical research settings, particularly for categorical responses. The following parameters were used for sampling: chain = 4, iter = 4000, warmup = 2000, thin = 1, and max_treedepth = 30. The performance and convergence diagnostics of the model, including the Rhat statistics and effective sample size, were also evaluated.^[19,20]^ The statistical software R (version 4.3.2; R Foundation for Statistical Computing, Vienna, Austria) and the RStan (version 2.32.3) package were used for statistical analyses.

## 3. Results

### 3.1. Patient characteristics

A total of 5160 patients were eligible to participate in this study. However, 5063 were excluded for various reasons: they were aged <20 years with a visual or other physical impairment, had cognitive impairments, had mental health diseases, had received psychotropic agents, had MAVs of >1, and reported that they had lost medications or did not bring all their medications to the hospital. Ultimately, 83 patients were included in this study. None of them reported not bringing along all of their prescription medications or having lost them. Table 1 presents patient characteristics and clinical information. Of these, 41% were male, with a median age of 66 years (IQR: 55–76 years). Almost half of the patients (53%) were aged ≥65 years. The median HbA1c level was 8.6% (IQR: 7.9%–9.5%). Concerning complications, 63%, 45%, and 9.6% had nephropathy, retinopathy, and proliferative retinopathy, respectively. None of the patients had macular edema. Thirty-four percent of patients had macrovascular complications. The median numbers of chronic diseases, OHAs, and nonhypoglycemic agents were 3 (IQR: 2–5), 2 (IQR: 2–3), and 5 (IQR: 3–9), respectively. Additionally, 60% of the patients concomitantly used insulin. Concerning adverse effects, 23% experienced hypoglycemia due to hypoglycemic agents. Sixty-three percent of patients had a family history of diabetes mellitus.

**Table 1:** Patient characteristics, clinical, and prescription information.

Patient characteristics and clinical information	
Male (%)	41
Age (yr)	66 (55–76)
20–64 yr (%)	47
≥65 yr (%)	53
Duration of diabetes (yr)	16 (9–22)
<10 yr (%)	27
10–19 yr (%)	37
≥20 yr (%)	36
BMI (kg/m^2^)	25.8 (23.1–29.4)
<25 kg/m^2^ (%)	43
≥25 kg/m^2^ (%)	57
HbA1C (%)	8.6 (7.9–9.5)
Complications of diabetes (%)	
Nephropathy	63
Retinopathy	45
Macrovascular disease	34
Number of chronic diseases	3 (2–5)
Total number of medications	8 (6–12)
Number of OHAs	2 (2–3)
Insulin therapy (%)	60
Number of nonhypoglycemic agents	5 (3–9)
Adverse events related hypoglycemic agent (%)	
Hypoglycemia	23
Others	6
Family history of diabetes (%)	63
Current drinking (%)	16
Dietary supplement (%)	17
Employed (%)	35
Living alone (%)	17
Marital status (%)	65
In-hospital prescription (%)	100
Prescription information of OHAs taken for more than 48 wk	
Total number of OHAs	137
MAV	0.97 (0.90–1.00)
Dosing period of the same OHA (wk)	101 (59–191)
Number of doses/d (%)	
Once	56
Twice	18
3 times	26
OHAs administered before meals (%)	18
Administration of OHAs (%)	
At breakfast	96
At lunch	28
At dinner	43
Types of OHAs (%)	
DPP-4 inhibitors	36
Metformin	31
SGLT2 inhibitors	9
α-glycosidase inhibitors	9
Sulfonylureas	9
Glinides	4

Data were expressed as the median and interquartile range, numbers, or percentages.

BMI = body mass index, DPP-4 = dipeptidyl peptidase 4, MAVs = medication adherence values, OHAs = oral hypoglycemic agents, SGLT2 = sodium-glucose cotransporter 2.

### 3.2. Prescription information for OHAs and MAVs

The patient had 137 OHAs. Detailed prescription information for all OHAs is presented in Table 1. The median MAVs were 0.97 (IQR: 0.90–1.00). Approximately 60% of all OHAs had MAVs of >0.93. An MAV of >0.86 ≤ 0.93 was observed in 13% of OHAs overall. The MAV was ≤0.86 in 27% of all OHAs. The median dosing period for the same OHA was 101 weeks (IQR: 59–191). The most commonly prescribed dose was 1 dose per day (56%). Furthermore, OHAs were commonly administered during breakfast (96%). Among the prescribed OHAs, dipeptidyl peptidase 4 (DPP-4) inhibitors were the most commonly used (36%), followed by metformin (31%).

### 3.3. Effect of MAVs on HbA1c

**Figure 1.**
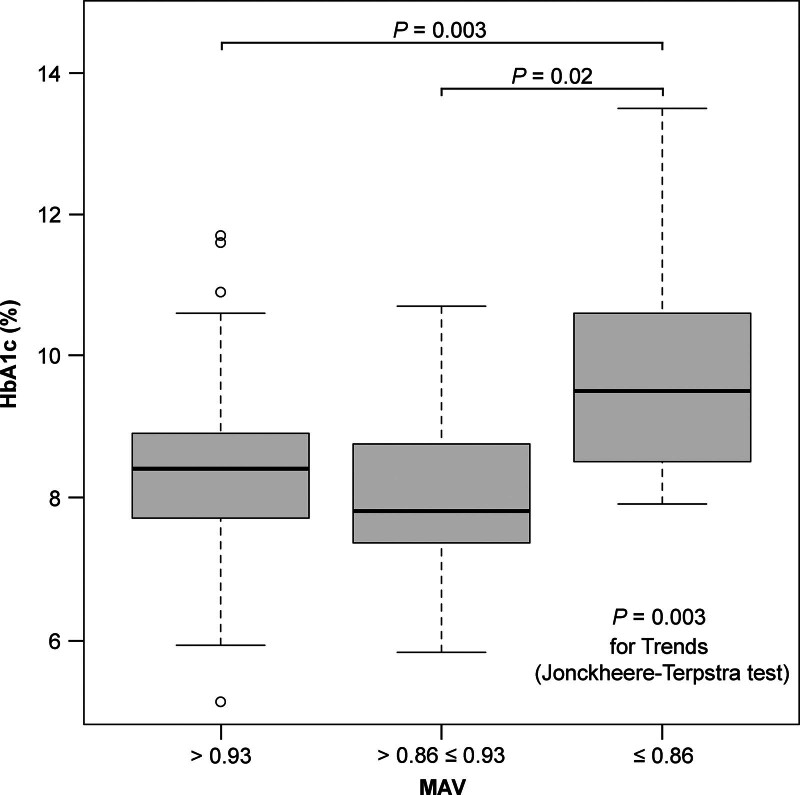
Changes in HbA1c with reduced medication adherence. Boxes and horizontal lines represent the interquartile range and median values, respectively. The upper and lower whiskers represent the high and low values of 1.5 × the interquartile range, respectively. The dots represent the outliers. *P* for trend analysis was based on the Jonckheere–Terpstra test. Pairwise comparisons were based on the Bonferroni correction. MAV = medication adherence value.

Figure 1 shows the distribution of MAVs into 3 categories (MAV: ≤0.86, >0.86 ≤ 0.93, and >0.93) and their corresponding changes in HbA1c levels. There was a trend toward higher HbA1c levels in the lower MAV group (*P* = .003). There was no statistically significant difference in HbA1c levels between MAV >0.93 and >0.86 ≤ 0.93 groups. However, HbA1c showed a significantly higher value in the MAV ≤0.86 group than in the MAV >0.93 (*P* = .003) and > 0.86 ≤ 0.93 (*P* = .02) groups.

### 3.4. Patient-related factors affecting MAVs

Based on the MAV results categorized into 3 groups and the corresponding HbA1c changes (Fig. 1), 2 groups of patients were formed: MAV ≤0.86 (indicating poor adherence) and MAV >0.86 (indicating good adherence). Table 2 shows patient-related factors associated with poor and good adherence. Poor and good adherence were used as the dependent variables in the logistic regression analysis, while variables and patient-related factors with *P* < .2 in the univariate analysis were used as the independent variables. Multicollinearity between the total number of medications and nonhypoglycemic agents was observed, as indicated by a VIF ≥5. A VIF of ≥5 suggests a potentially strong correlation, signifying multicollinearity. Therefore, we use 2 models for this analysis. In model 1, BMI, number of chronic diseases, total number of medications, number of OHAs, adverse events related to hypoglycemic agents (excluding hypoglycemia), and family history of diabetes were used as independent variables. In model 2, BMI, number of chronic diseases, number of OHAs, number of nonhypoglycemic agents, adverse events related to hypoglycemic agents (excluding hypoglycemia), and family history of diabetes were used as independent variables in the multivariate analysis. As shown in Table 2, In model 1, logistic regression analysis demonstrated a significant correlation between poor adherence, total number of medications, and family history of diabetes (*P* < .05). Meanwhile, model 2 identified a significant correlation between poor adherence and the number of nonhypoglycemic agents and a family history of diabetes (*P* < .05) (Table 2).

**Table 2:** Patient-related factors of medication adherence as identified via logistic regression analysis.

Variable	MAVs		Univariate model		Multivariate model 1		Multivariate model 2	
	≤0.86 (n = 22)	>0.86 (n = 61)	OR (95% CI)	*P* value	OR (95% CI)	*P* value	OR (95% CI)	*P* value
Male (%)	32	44	1.70 (0.61–4.76)	.31				
Age (20–64, ≥65 yr)			0.66 (0.25–1.76)	.41				
20–64 yr (%)	55	44						
≥65 yr (%)	45	56						
Duration of diabetes (<10, 10–19, ≥20 yr)			0.66 (0.35–1.24)	.20				
<10 yr (%)	36.4	23						
10–19 yr (%)	36.4	38						
≥20 yr (%)	27.2	39						
BMI (<25, ≥25 kg/m^2^)			2.58 (0.89–7.48)	.08				
<25 kg/m^2^ (%)	27	49						
≥25 kg/m^2^ (%)	73	51						
Complications of diabetes (%)								
Nephropathy	64	62	1.01 (0.37–2.80)	.98				
Retinopathy	50	41	1.40 (0.53–3.73)	.50				
Macrovascular disease	32	34	0.89 (0.31–2.52)	.83				
Number of chronic diseases	2.5 (2–3.8)	3 (2–5)	0.77 (0.58–1.02)	.06				
Total number of medication	7.5 (5–9)	9 (7–12)	0.89 (0.79–1.02)	.09	0.86 (0.75–0.99)	.03		
Number of OHAs	2 (2–3)	2 (2–3)	1.46 (0.84–2.53)	.18				
Insulin therapy (%)	55	62	0.73 (0.27–1.95)	.53				
Number of nonhypoglycemic agents	4 (2.3–6.8)	6 (4–9)	0.88 (0.77–1.01)	.07			0.85 (0.73–0.98)	.02
Adverse events related hypoglycemic agent (%)								
Hypoglycemia	27	21	1.38 (0.45–4.25)	.57				
Others	14	5	3.05 (0.57–16.40)	.19				
Family history of diabetes (%)	50	67	0.46 (0.17–1.26)	.13	0.34 (0.11–0.99)	.04	0.32 (0.11–0.95)	.04
Current drinking (%)	14	16	0.81 (0.20–3.24)	.76				
Dietary supplement (%)	18	16	1.13 (0.32–4.07)	.85				
Employed (%)	36	34	1.09 (0.39–3.01)	.87				
Living alone (%)	14	18	0.72 (0.18–2.86)	.64				
Marital status (%)	68	64	1.21 (0.43–3.41)	.72				

Data were expressed as the median and interquartile range or percentages. Model 1: BMI, number of chronic diseases, total number of medications, number of OHAs, adverse events related to hypoglycemic agents (excluding hypoglycemia), family history of diabetes. Model 2: BMI, number of chronic diseases, number of OHAs, number of nonhypoglycemic agents, adverse events related to hypoglycemic agents (excluding hypoglycemia), family history of diabetes.

BMI = body mass index, CI = confidence interval, MAVs = medication adherence values, OHAs = oral hypoglycemic agents, OR = odds ratio.

In addition to the traditional logistic regression analysis, a Bayesian analysis was conducted using RStan to further explore the factors influencing medication adherence. Tables 1 and 2, Supplemental Digital Content, http://links.lww.com/MD/M122, http://links.lww.com/MD/M123, show the summary of the statistical values for each variable. The estimated effects represent the association between each explanatory variable and the probability of medication adherence after controlling for other variables in the model. Convergence diagnostics for models 1 and 2 indicated satisfactory mixing and convergence of the chains, with all Rhat values being 1. The effective sample size (n_eff) of each factor was well above the generally accepted threshold, indicating a reliable estimation of the posterior distributions. In models 1 and 2, a family history of diabetes was significantly associated with medication adherence (estimated effect: −1.34, 95% confidence interval: −2.63 to −0.12).

### 3.5. Medication-related factors affecting MAVs

Table 3 shows the medication-related factors of medication adherence identified using the logistic regression analysis. Poor and good medication adherence were used as dependent variables in these analyses, while characteristics related to medication that had a *P* <.2 in the univariate analysis were used as independent variables. Logistic regression analyses showed that none of the medication-related factors could independently explain poor adherence. Similarly, in the Bayesian analysis, none of the medication-related factors were significantly associated with medication adherence.

**Table 3:** Medication-related factors of medication adherence as identified via logistic regression analysis.

Variable	MAVs		Univariate model	
	≤0.86	>0.86	OR (95% CI)	*P* value
Total number of OHAs	31	106		
Dosing period of the same OHA (wk)	102 (53–168)	99 (61–198)	0.99 (0.99–1.00)	.62
Number of doses/d (%)			1.49 (0.95–2.35)	.09
Once	42	60		
Twice	23	16		
3 times	35	24		
OHAs administered before meals (%)	23	17	1.43 (0.53–3.81)	.48
Administration of OHAs (%)				
At breakfast	97	96	0.88 (0.09–8.80)	.92
At lunch	39	25	1.82 (0.78–4.25)	.16
At dinner	58	39	2.16 (0.96–4.88)	.06
Types of OHAs (%)				
DPP-4 inhibitors	23	40	0.44 (0.18–1.12)	.09
Metformin	39	29	1.53 (0.66–3.52)	.32
Sulfonylureas	13	8	1.60 (0.46–5.59)	.46
α-glycosidase inhibitors	13	8	1.81 (0.51–6.49)	.36
SGLT2 inhibitors	10	9	1.03 (0.27–4.00)	.97
Glinides	3	5	0.67 (0.08–5.99)	.72

Data were expressed as the median and interquartile range, numbers, or percentages.

CI = confidence interval, DPP-4 = dipeptidyl peptidase 4, MAVs = medication adherence values, OHAs = oral hypoglycemic agents, SGLT2 = sodium-glucose cotransporter 2, OR = odds ratio.

We then performed multiple regression analyses with each MAV as the dependent variable to further investigate the medication-related factors and medication adherence. Table 4 shows the medication-related factors associated with MAVs. Multiple regression analyses were performed with each MAV as the dependent variable and medication-related factors with *P* < .2 in the univariate analysis as the independent variables. Multicollinearity was observed between the number of doses per day and the administration of OHAs at dinner. with a VIF of ≥5. Consequently, we use 2 models for this analysis. In model 1, the number of doses per day, administration of OHAs at lunch, use of DPP-4 inhibitors, and use of α-glycosidase inhibitors were used as independent variables. In model 2, the administration of OHAs at lunch and dinner, the use of DPP-4 inhibitors, and the use of α-glycosidase inhibitors were used as independent variables in multivariate analysis. In model 1, multiple regression analysis revealed a significant correlation (*P* < .05) between the number of dosages per day and MAVs, while in model 2, the administration of OHAs at lunch was significantly correlated with MAVs (*P* < .05) (Table 4). In the Bayesian analysis, none of the medication-related factors were significantly associated with MAVs (Tables 3 and 4, Supplemental Digital Content, http://links.lww.com/MD/M125, http://links.lww.com/MD/M127).

**Table 4:** Medication-related factors of medication adherence as identified via multiple regression analysis.

Variable	Univariate model			Multivariate model 1			Multivariate model 2		
	*β*	SE	*P* value	*β*	SE	*P* value	*β*	SE	*P* value
Dosing period of the same OHA (wk)	−0.00002	0.0001	.79						
Number of doses/d	−0.02785	0.01066	.01	−0.0276	0.0107	.01			
OHAs administered before meals	−0.02814	0.02414	.25						
Administration of OHAs									
At breakfast	−0.01295	0.0558	.82						
At lunch	−0.04991	0.0204	.02				−0.0499	0.02	.02
At dinner	−0.03952	0.01872	.04						
Types of OHAs									
DPP-4 inhibitors	0.0285	0.01939	.14						
Metformin	−0.01631	0.02014	.42						
Sulfonylureas	−0.0242	0.0319	.45						
α-glycosidase inhibitors	−0.06512	0.03266	.04						
SGLT2 inhibitors	0.01554	0.03194	.63						
Glinides	0.03118	0.04571	.49						

Model 1: number of doses/d, administration of OHAs at lunch, DPP-4 inhibitors, α-glycosidase inhibitors. Model 2: administration of OHAs at lunch, administration of OHAs at dinner, DPP-4 inhibitors, α-glycosidase inhibitors.

*β* = standard regression coefficient, DPP-4 = dipeptidyl peptidase 4, OHAs = oral hypoglycemic agents, SE = standard error, SGLT2 = sodium-glucose cotransporter 2.

## 4. Discussion

The results of this study revealed a connection between HbA1c and the continued use of OHAs, as evaluated by pill counting over 48 weeks. In particular, HbA1c levels were significantly higher in patients who missed taking their OHAs more than once a week for over 48 weeks than in those who took their OHAs continuously. Furthermore, these data suggest that poor medication adherence is associated with the total number of medications, number of nonhypoglycemic agents, and family history of diabetes mellitus. The results of this study provide a more accurate assessment of HbA1c changes and factors affecting medication adherence in T2DM patients who self-administer their medications. This assessment also considers instances of discontinued temporality owing to surgery or tests, all in an environment where patients exhibit low psychological bias. To the best of our knowledge, this is the most reliable analysis to examine the relationship between HbA1c changes and OHA medication adherence in patients with T2DM over 48 weeks.

According to the findings of some earlier investigations, glycemic control and medication adherence are related.^[21–24]^ Although different from our methods, these investigations employed either the medication possession ratio or a questionnaire-based approach. Patients with poor adherence to OHAs, as determined by the percentage of medication taken on prescription for up to 365 days, had lower HbA1c reductions than those taking treatment as advised.^[21,22]^ Therefore, our previous study on the use of medications for >24 weeks did not show a significant correlation between medication adherence and HbA1c levels,^[14]^ revealing that persistently poor adherence for >48 weeks can have an impact on HbA1c levels.

Our study identified patient-related factors affecting medication adherence, including the total number of medications, nonhypoglycemic agents, and a family history of diabetes. The 2 factors exhibited a high correlation coefficient because multicollinearity was observed between the total number of medications and nonhypoglycemic agents. The number of nonhypoglycemic agents can be related to the number and severity of chronic diseases. Patients with a higher number of comorbidities and more provider follow-up visits had higher adherence to medication according to a previous systematic review that sought to identify the factors that affect nonadherence in patients with dyslipidemia, a chronic disease such as diabetes.^[25]^ The greater the number of comorbidities or nonhypoglycemic agents, the greater their communication with their doctors, and the greater their understanding of the severity of their condition. This may have led to improved medication adherence. However, the knowledge of disease significance levels may vary based on comorbidities. A systematic review examining the characteristics affecting the adherence of adult patients with chronic physical diseases found that depression had a negative effect on adherence.^[26]^ However, due to the small number of patients in this investigation, an analysis according to comorbidities was not performed. Therefore, a detailed study on each comorbidity is required.

Previous research has documented the connections between adherence and a family history of diabetes.^[27]^ It has also been found that a family history of diabetes-related chronic conditions like hypertension and cardiovascular disease is associated with improved adherence.^[28,29]^ Individuals who have comorbidities with cardiovascular risk factors are more likely to be aware of their increased risk; as a result, they are more likely to follow a treatment plan.^[28]^ According to the Interact Consortium study, individuals with a family history of diabetes potentially engage in healthy behaviors and can significantly benefit from such lifestyle interventions.^[30]^ A previous study on a family history of diabetes mellitus and adherence found a significant correlation between a family history of diabetes mellitus and frequent exercise and adherence to a balanced diet.^[31]^ In addition, among patients with T2DM, physical activity adherence was higher in those with a positive family history of diabetes than others.^[32]^ Thus, this behavior change can impact adherence.

Furthermore, our study identified medication-related factors affecting medication adherence, including the number of dosages per day and administration of OHAs at lunch. According to a review that looked at how regimen simplification strategies affected medication adherence, once-daily dosing, fixed-dose combinations, and a combination of both were effective regimen simplification strategies for boosting medication adherence.^[33]^ Once-daily medicine may reduce the likelihood of treatment noncompliance in patients with chronic cardiovascular disease by approximately 50%, according to a previous meta-analysis.^[34]^ Previous studies in patients with diabetes have shown that reducing the frequency of medication use may reduce the number of patients with poor medication adherence.^[35,36]^ The dosing frequency has been linked to medication adherence in several disorders, according to previous studies.^[37,38]^ As a result, a modified dosing schedule and a switch to a once-daily dosage may increase adherence. Therefore, it is important to assess and optimize the dosage for each patient with diabetes. Poor lunchtime medication adherence may be associated with going out to work. Due to time constraints during lunch breaks and the lack of OHAs when leaving the house, medication adherence could be negatively affected.

Herein, discrepancies were observed between the results obtained from the traditional logistic regression analysis and those from the Bayesian analysis using RStan. Logistic regression analysis showed statistically significant associations between variables such as the total number of medications, use of nonhypoglycemic agents, dosages per day, and the administration of OHAs at lunch as well as medication adherence. However, the Bayesian methods did not exhibit the same level of significance. This divergence requires cautious consideration of the inherent differences between the 2 analytical approaches and their underlying assumptions. The influence of priors, a central aspect of Bayesian analysis, can significantly affect outcomes, particularly in cases involving limited data or subtle effects. In addition, the sensitivity of the model to sample size and data variability might have contributed to the various results, thereby underscoring the need for caution when interpreting and generalizing the findings. Bayesian analysis, with its comprehensive estimation of uncertainty and model robustness, would explain the less pronounced effects observed compared to the results of the logistic regression analysis. The contrasting results of this study underscore the importance of considering multiple analytical approaches in research. Future investigations should use different statistical methods to corroborate these findings, and additional data collection can provide more comprehensive insights.

This study has some limitations. First, although the patients were instructed to bring all their prescriptions with them from home before and throughout the hospitalization period, we could only believe what they said. Patients who might have been overestimated were excluded if their MAV was >1. We had no choice but to rely on the self-reports of patients. Second, we included hospitalized patients and those who had been taking the same OHA for at least 48 weeks before hospitalization. Therefore, excluding individuals who had not been hospitalized or who had not been on the same OHA for 48 weeks might be biased. Finally, the medical insurance systems differ between Japan and other countries. Under Japan’s universal health insurance system, all Japanese are covered by public medical insurance. This system difference could be the reason for variations in the results of other studies.

## 5. Conclusion

Our study reaffirms the important role of medication adherence in the management of type 2 diabetes. The results showed that skipping OHAs more than once a week was associated with a negative impact on HbA1c levels, thereby underlining the significance of consistent medication intake. Furthermore, our findings emphasize that several factors, including the total number of medications, use of nonhypoglycemic agents, family history of diabetes, dosing frequency, and timing of medication administration, particularly at lunch, affect medication adherence. These findings have several important clinical implications. Healthcare providers should prioritize patient education and support to address these factors and improve medication adherence. Strategies such as simplifying dosing regimens and engaging patients in shared decision making can help achieve better outcomes. Working collaboratively, healthcare providers and patients can strive to improve glycemic control, ultimately reducing the risk of complications associated with T2DM. This study sheds light on these factors. However, it is essential to acknowledge its limitations, including its reliance on self-reported medication intake. Future research based on our findings should explore interventions to enhance adherence and investigate the long-term impact of improved medication adherence on patient outcomes. Overall, our study underscores the need for a patient-centered approach to diabetes management that focuses on optimizing medication adherence to improve health outcomes.

## Acknowledgments

The authors would like to thank Enago (www.enago.jp) for the English language review.

## Authorship

All named authors meet the International Committee of Medical Journal Editors criteria for authorship of this article, take responsibility for the integrity of the work as a whole, and have approved this version of the manuscript to be published.

## Author contributions

**Conceptualization:** Megumi Shiomi, Tesshu Takada, Katsuya Otori, Kiyoshi Shibuya.

**Formal analysis:** Megumi Shiomi.

**Validation:** Megumi Shiomi.

**Writing—original draft:** Megumi Shiomi.

**Writing—review & editing:** Tesshu Takada, Katsuya Otori, Kiyoshi Shibuya.

## Supplementary Material









## References

[1] Cramer JA. A systematic review of adherence with medications for diabetes. Diabetes Care. 2004;27:1218–24. 15111553 10.2337/diacare.27.5.1218

[2] Egede LE Gebregziabher M Echols C. Longitudinal effects of medication nonadherence on glycemic control. Ann Pharmacother. 2014;48:562–70. 24586059 10.1177/1060028014526362

[3] Ho PM Rumsfeld JS Masoudi FA. Effect of medication nonadherence on hospitalization and mortality among patients with diabetes mellitus. Arch Intern Med. 2006;166:1836–41. 17000939 10.1001/archinte.166.17.1836

[4] Lam WY Fresco P. Medication adherence measures: an overview. Biomed Res Int. 2015;2015:217047. 26539470 10.1155/2015/217047 PMC4619779

[5] Tunceli K Zhao C Davies MJ. Factors associated with adherence to oral antihyperglycemic monotherapy in patients with type 2 diabetes. Patient Prefer Adherence. 2015;9:191–7. 25670888 10.2147/PPA.S71346 PMC4315552

[6] Kirkman MS Rowan-Martin MT Levin R. Determinants of adherence to diabetes medications: findings from a large pharmacy claims database. Diabetes Care. 2015;38:604–9. 25573883 10.2337/dc14-2098 PMC4370331

[7] Curkendall SM Thomas N Bell KF. Predictors of medication adherence in patients with type 2 diabetes mellitus. Curr Med Res Opin. 2013;29:1275–86. 23815104 10.1185/03007995.2013.821056

[8] Garcia ML Castañeda SF Allison MA. Correlates of low-adherence to oral hypoglycemic medications among Hispanic/Latinos of Mexican heritage with type 2 diabetes in the United States. Diabetes Res Clin Pract. 2019;155:107692. 30954512 10.1016/j.diabres.2019.04.007 PMC9494711

[9] Krass I Schieback P Dhippayom T. Adherence to diabetes medication: a systematic review. Diabet Med. 2015;32:725–37. 25440507 10.1111/dme.12651

[10] Farmer KC. Methods for measuring and monitoring medication regimen adherence in clinical trials and clinical practice. Clin Ther. 1999;21:1074–90; discussion 1073. 10440628 10.1016/S0149-2918(99)80026-5

[11] van Onzenoort HA Verberk WJ Kessels AG. Assessing medication adherence simultaneously by electronic monitoring and pill count in patients with mild-to-moderate hypertension. Am J Hypertens. 2010;23:149–54. 19927136 10.1038/ajh.2009.207

[12] Takahara M Shiraiwa T Ogawa N. Clinical backgrounds associated with discrepancy between subjective and objective assessments of medication adherence in Japanese type 2 diabetic patients. Diabetol Int. 2016;7:398–403. 30603292 10.1007/s13340-016-0265-z PMC6224975

[13] Niechciał E Acerini CL Chiesa ST.; Adolescent Type 1 Diabetes Cardio-Renal Intervention Trial (AdDIT) Study Group. Medication adherence during adjunct therapy with statins and ACE inhibitors in adolescents with type 1 diabetes. Diabetes Care. 2020;43:1070–6. 32108022 10.2337/dc19-0884 PMC7282885

[14] Shiomi M Kurobuchi M Tanaka Y. Pill counting in the determination of factors affecting medication adherence in patients with type 2 diabetes: a retrospective observational study. Diabetes Ther. 2021;12:1993–2005. 34120302 10.1007/s13300-021-01091-1 PMC8266921

[15] Haneda M Utsunomiya K Koya D. A new classification of diabetic nephropathy 2014: a report from the Joint Committee on Diabetic Nephropathy. Clin Exp Nephrol. 2015;19:1–5. 25527479 10.1007/s10157-014-1057-z

[16] Wilkinson CP Ferris FL Klein RE.; Global Diabetic Retinopathy Project Group. Proposed international clinical diabetic retinopathy and diabetic macular edema disease severity scales. Ophthalmology. 2003;110:1677–82. 13129861 10.1016/S0161-6420(03)00475-5

[17] Kanda Y. Investigation of the freely available easy-to-use software “EZR” for medical statistics. Bone Marrow Transplant. 2013;48:452–8. 23208313 10.1038/bmt.2012.244 PMC3590441

[18] Bhattacharyya A Pal S Mitra R. Applications of Bayesian shrinkage prior models in clinical research with categorical responses. BMC Med Res Methodol. 2022;22:126. 35484507 10.1186/s12874-022-01560-6 PMC9046716

[19] Luo Y Jiao H. Using the Stan program for Bayesian item response theory. Educ Psychol Meas. 2018;78:384–408. 30140099 10.1177/0013164417693666 PMC6096466

[20] Nishio M Akasaka T Sakamoto R. Bayesian statistical model of item response theory in observer studies of radiologists. Acad Radiol. 2020;27:e45–54. 31147237 10.1016/j.acra.2019.04.014

[21] Farmer AJ Rodgers LR Lonergan M. Adherence to oral glucose-lowering therapies and associations with 1-year HbA1c: a retrospective cohort analysis in a large primary care database. Diabetes Care. 2016;39:258–63. 26681714 10.2337/dc15-1194 PMC4894467

[22] Gordon J McEwan P Idris I. Treatment choice, medication adherence and glycemic efficacy in people with type 2 diabetes: a UK clinical practice database study. BMJ Open Diabetes Res Care. 2018;6:e000512. 10.1136/bmjdrc-2018-000512 PMC5942418 29755756

[23] Pourhabibi N Mohebbi B Sadeghi R. Factors associated with treatment adherence to treatment among in patients with type 2 diabetes in Iran: a cross-sectional study. Front Public Health. 2022;10:976888. 36407991 10.3389/fpubh.2022.976888 PMC9667890

[24] Abdullah NF Khuan L Theng CA. Prevalence and reasons influenced medication non-adherence among diabetes patients: a mixed-method study. J Diabetes Metab Disord. 2022;21:1669–78. 36404839 10.1007/s40200-022-01118-9 PMC9672180

[25] Lopes J Santos P. Determinants of non-adherence to the medications for dyslipidemia: a systematic review. Patient Prefer Adherence. 2021;15:1853–71. 34465984 10.2147/PPA.S319604 PMC8403077

[26] Sahoo J Mohanty S Kundu A. Medication adherence among patients of type II diabetes mellitus and its associated risk factors: a cross-sectional study in a Tertiary Care Hospital of Eastern India. Cureus. 2022;14:e33074. 36721541 10.7759/cureus.33074 PMC9883658

[27] Kim H Kim HS Bowman JD. Comparing diabetic patient characteristics related to stated medication adherence in a Rural vs. Urban Community in Korea. J Clin Pharm Ther. 2016;41:40–6. 26714628 10.1111/jcpt.12344

[28] Mazzaglia G Ambrosioni E Alacqua M. Adherence to antihypertensive medications and cardiovascular morbidity among newly diagnosed hypertensive patients. Circulation. 2009;120:1598–605. 19805653 10.1161/CIRCULATIONAHA.108.830299

[29] Choi HY Oh IJ Lee JA. Factors affecting adherence to antihypertensive medication. Korean J Fam Med. 2018;39:325–32. 30384549 10.4082/kjfm.17.0041 PMC6250947

[30] Scott RA Langenberg C Sharp SJ.; InterAct Consortium. The link between family history and risk of type 2 diabetes is not explained by anthropometric, lifestyle or genetic risk factors: the EPIC-InterAct study. Diabetologia. 2013;56:60–9. 23052052 10.1007/s00125-012-2715-x PMC4038917

[31] Choi J Choi JY Lee SA. Association between family history of diabetes and clusters of adherence to healthy behaviors: cross-sectional results from the Health Examinees-Gem (HEXA-G) Study. BMJ Open. 2019;9:e025477. 10.1136/bmjopen-2018-025477 PMC6588964 31209083

[32] Parajuli J Saleh F Thapa N. Factors associated with nonadherence to diet and physical activity among Nepalese type 2 diabetes patients; a cross sectional study. BMC Res Notes. 2014;7:758. 25344089 10.1186/1756-0500-7-758 PMC4230343

[33] Elnaem MH Irwan NA Abubakar U. Impact of medication regimen simplification on medication adherence and clinical outcomes in patients with long-term medical conditions. Patient Prefer Adherence. 2020;14:2135–45. 33173282 10.2147/PPA.S268499 PMC7646472

[34] Caldeira D Vaz-Carneiro A Costa J. The impact of dosing frequency on medication adherence in chronic cardiovascular disease: systematic review and meta-analysis. Rev Port Cardiol. 2014;33:431–7. 25070671 10.1016/j.repc.2014.01.013

[35] Paes AH Bakker A Soe-Agnie CJ. Impact of dosage frequency on patient compliance. Diabetes Care. 1997;20:1512–7. 9314626 10.2337/diacare.20.10.1512

[36] Ayoub D Mroueh L El-Hajj M. Evaluation of antidiabetic medication adherence in the Lebanese population: development of the Lebanese diabetes medication adherence scale. Int J Pharm Pract. 2019;27:468–76. 31264750 10.1111/ijpp.12558

[37] Laliberté F Bookhart BK Nelson WW. Impact of once-daily versus twice-daily dosing frequency on adherence to chronic medications among patients with venous thromboembolism. Patient. 2013;6:213–24. 23857628 10.1007/s40271-013-0020-5 PMC3751276

[38] Oh CK Bang JB Kim SJ. Improvement of medication adherence with simplified once-daily immunosuppressive regimen in stable kidney transplant recipients: a prospective cohort study. Asian J Surg. 2020;43:660–7. 31353239 10.1016/j.asjsur.2019.07.011

