# Supplementary material for: Frequency of missed doses and its effects on the regulation of glucose levels in patients with type 2 diabetes: A retrospective analysis
Source: Medicine (Baltimore). 2024 Apr 12;103(15):e37711. doi: 10.1097/MD.0000000000037711 (PMC11018172; doi:10.1097/MD.0000000000037711)
Supplement: Supplementary file 1 [file medi-103-e37711-s001.docx]

| Variable | Estimated effect | SD | 2.5% | 25% | 50% | 75% | 97.5% | n_eff | Rhat |
| --- | --- | --- | --- | --- | --- | --- | --- | --- | --- |
| BMI (<25, ≥25 kg/m^2^) | 1.21 | 0.65 | –0.01 | 0.77 | 1.19 | 1.65 | 2.56 | 5259 | 1 |
| Number of chronic diseases | −0.22 | 0.28 | –0.77 | –0.40 | –0.21 | –0.03 | 0.30 | 5083 | 1 |
| Total number of medications | –0.15 | 0.13 | –0.40 | –0.23 | –0.15 | –0.06 | 0.09 | 4619 | 1 |
| Number of OHAs | 0.51 | 0.35 | –0.16 | 0.27 | 0.50 | 0.74 | 1.22 | 6680 | 1 |
| Adverse events related to hypoglycemic agents  (excluding hypoglycemia) | 2.01 | 1.17 | –0.18 | 1.23 | 1.98 | 2.76 | 4.44 | 6619 | 1 |
| Family history of diabetes | –1.34 | 0.63 | –2.63 | –1.75 | –1.32 | –0.91 | –0.12 | 6316 | 1 |

**Supplemental Table 1. Posterior summary of model coefficients for patient-related factors and medication adherence in model 1**

BMI, body mass index; OHAs, oral hypoglycemic agents; SD, standard deviation.

Model 1: BMI, number of chronic diseases, total number of medications, number of OHAs, adverse events related to hypoglycemic agents (excluding hypoglycemia), and family history of diabetes.
