# Supplementary material for: Frequency of missed doses and its effects on the regulation of glucose levels in patients with type 2 diabetes: A retrospective analysis
Source: Medicine (Baltimore). 2024 Apr 12;103(15):e37711. doi: 10.1097/MD.0000000000037711 (PMC11018172; doi:10.1097/MD.0000000000037711)
Supplement: Supplementary file 2 [file medi-103-e37711-s002.docx]

| Variable | Estimated effect | SD | 2.5% | 25% | 50% | 75% | 97.5% | n_eff | Rhat |
| --- | --- | --- | --- | --- | --- | --- | --- | --- | --- |
| BMI (<25, ≥25 kg/m^2^) | 1.23 | 0.67 | –0.01 | 0.77 | 1.21 | 1.67 | 2.62 | 5654 | 1 |
| Number of chronic diseases | –0.26 | 0.28 | –0.83 | –0.44 | –0.25 | –0.07 | 0.27 | 5304 | 1 |
| Number of OHAs | 0.40 | 0.35 | –0.27 | 0.17 | 0.39 | 0.63 | 1.09 | 6572 | 1 |
| Number of nonhypoglycemic agents | 0.12 | 0.13 | –0.38 | –0.21 | –0.12 | –0.04 | 0.12 | 4724 | 1 |
| Adverse events related to hypoglycemic agents  (excluding hypoglycemia) | 1.98 | 1.18 | –0.28 | 1.19 | 1.95 | 2.73 | 4.42 | 7147 | 1 |
| Family history of diabetes | –1.33 | 0.62 | –2.58 | –1.75 | –1.32 | –0.91 | –0.14 | 6986 | 1 |

**Supplemental Table 2. Posterior summary of model coefficients for patient-related factors and medication adherence in model 2**

BMI, body mass index; OHAs, oral hypoglycemic agents; SD, standard deviation.

Model 2: BMI, number of chronic diseases, number of OHAs, number of nonhypoglycemic agents, adverse events related to hypoglycemic agents (excluding hypoglycemia), and family history of diabetes.
