# Supplementary material for: Frequency of missed doses and its effects on the regulation of glucose levels in patients with type 2 diabetes: A retrospective analysis
Source: Medicine (Baltimore). 2024 Apr 12;103(15):e37711. doi: 10.1097/MD.0000000000037711 (PMC11018172; doi:10.1097/MD.0000000000037711)
Supplement: Supplementary file 3 [file medi-103-e37711-s003.docx]

| Variable | Estimated effect | SD | 2.5% | 25% | 50% | 75% | 97.5% | n_eff | Rhat |
| --- | --- | --- | --- | --- | --- | --- | --- | --- | --- |
| Number of doses/day | –0.02 | 0.02 | –0.06 | –0.03 | –0.02 | 0 | 0.02 | 1744 | 1 |
| Administration of OHAs  at lunch | –0.01 | 0.04 | –0.08 | –0.04 | –0.01 | 0.01 | 0.05 | 2342 | 1 |
| DPP-4 inhibitors | 0 | 0.02 | –0.05 | –0.02 | 0 | 0.01 | 0.05 | 2331 | 1 |
| α-glycosidase inhibitors | –0.04 | 0.03 | –0.11 | –0.07 | –0.04 | –0.02 | 0.02 | 2873 | 1 |

**Supplemental Table 3. Posterior summary of model coefficients for medication-related factors and each medication adherence**

**in model 1**

DPP4, dipeptidyl peptidase 4; OHAs, oral hypoglycemic agents; SD, standard deviation.

Model 1: number of doses/day, administration of OHAs at lunch, DPP-4 inhibitors, and α-glycosidase inhibitors.
